# Determination of *HER2* amplification in primary breast cancer using dual-colour chromogenic *in situ* hybridization is comparable to fluorescence *in situ* hybridization: a European multicentre study involving 168 specimens

**DOI:** 10.1111/j.1365-2559.2010.03503.x

**Published:** 2010-03

**Authors:** Tomás García-Caballero, Dorthe Grabau, Andrew R Green, John Gregory, Arno Schad, Elke Kohlwes, Ian O Ellis, Sarah Watts, Jens Mollerup

**Affiliations:** Department of Morphological Sciences, School of Medicine-University Clinical Hospital, University of Santiago de CompostelaSantiago de Compostela, Spain; 1 Division of Pathology and Cytology, University Hospital of LundLund, Sweden; 2 Division of Pathology, School of Molecular Medical Sciences, University of NottinghamNottingham; 3 University Hospital Birmingham NHS Foundation TrustBirmingham, UK; 4 Institute of Pathology, Johannes Gutenberg University HospitalMainz, Germany; 5 Dako Denmark A/SGlostrup, Denmark

**Keywords:** breast cancer, CEN-17, CISH, FISH, *HER2*, *HER2* amplification, *in situ* hybridization

## Abstract

García-Caballero T, Grabau D, Green A R, Gregory J, Schad A, Kohlwes E, Ellis I O, Watts S & Mollerup J (2010) *Histopathology***56,** 472–480 **Determination of *HER2* amplification in primary breast cancer using dual-colour chromogenic *in situ* hybridization is comparable to fluorescence *in situ* hybridization: a European multicentre study involving 168 specimens**

**Aims:**

Fluorescence *in situ* hybridization (FISH) can be used to reveal several genomic imbalances relevant to proper cancer diagnosis and to the correct treatment regime. However, FISH requires expensive and advanced fluorescence microscopes in addition to expertise in fluorescence microscopy. To determine whether a newly developed dual-colour chromogenic *in situ* hybridization (CISH) method is a suitable alternative to FISH, we analysed the human epidermal growth factor receptor 2 gene *(HER2)* amplification level of 168 breast cancer specimens using dual-colour CISH and FISH and compared the results.

**Methods and results:**

We found 100% agreement between HER2 status determined by FISH and dual-colour CISH. Furthermore, we observed that the time used to score slides was significantly reduced by 28% in dual-colour CISH compared with the FISH protocol. Concordance between HER2 protein status and dual-colour CISH or FISH was equally good with an overall agreement of 96.8%. Correlation between the *HER2*/centromere 17 gene ratios obtained with dual-colour CISH and FISH was highly significant with an overall correlation coefficient (ρ) of 0.96.

**Conclusions:**

We conclude that dual-colour CISH and bright field microscopy are excellent alternatives to FISH when analysing the HER2 status of primary breast cancer.

## Introduction

Fluorescence *in situ* hybridization (FISH) analysis is based on specific recognition of denatured target DNA sequences by fluorescent labelled sequence pairing probes. Gene amplifications, deletions, translocations as well as chromosomal copy number changes are among the types of genetic aberrations that can be detected by FISH. It is therefore a powerful technique used to reveal known genetic alterations relevant to cancer diagnosis, prognosis and correct treatment strategies.

The large potential for diagnostic and prognostic use of FISH is not implemented in all routine pathological laboratories. This could be due to disadvantages previously put forward,^1^–^4^ which include certain fixatives interfering with fluorescence detection and limited community experience with tissue-based FISH. Furthermore, the evaluation of tissue morphology through FISH is challenging, and the fluorescence signal fades relatively quickly, making long-term archiving of slides impossible. Comparisons of FISH with chromogenic *in situ* hybridization (CISH) have revealed several advantages with the use of CISH.^2^,^3^ The CISH technique is a simple extension of the FISH protocol that allows bright field microscopic evaluation of slides with the additional benefit that morphological features can be observed, and that archiving of slides is possible.

Conversion of the FISH to a CISH signal is achieved using fluorochrome- or hapten-specific antibodies as an extension to the FISH probes. These antibodies are conjugated to an enzymatic marker such as horseradish peroxidase (HRP) or alkaline phosphatase (AP). These enzymes are visualized by the addition of suitable substrates and the chromogenic signals can be quantified using bright field microscopy. Until now the CISH technique has mainly been done using one colour, although two dual-colour protocols have been developed and published.^5^,^6^ A major advantage of dual-colour *in situ* hybridization (ISH) is that a reference probe can be utilized, making it easier, faster and more accurate to distinguish true gene amplifications from chromosomal aneuploidy.^7^

One major application of FISH in routine pathology is the determination of human growth factor receptor 2 (HER2) status in primary breast cancer. Overexpression of HER2 has been documented in approximately 18–23% of human breast cancers^8^ and is considered a poor prognostic marker.^9^ Clinical Phase III studies of trastuzumab (Herceptin®; Genentech, San Francisco, CA, USA) in breast cancer have shown a great benefit, particularly for patients with tumours with HER2 gene (*HER2*) amplification.^10^–^12^ Current recommendations of the American Society of Clinical Oncology/College of American Pathologists (ASCO/CAP) include determination of HER2 status in all invasive breast cancers using immunohistochemistry or ISH.^8^

In this study, we present the data from a pan-European HER2 assessment study involving five different laboratories. We compared the results obtained with FISH and a newly available dual-colour CISH assay for the determination of HER2 status in 168 cases of primary breast cancer.

## Methods

### Tumour specimens

Routine formalin-fixed paraffin-embedded breast cancer specimens from 168 patients were included in this study. From each of four pathological laboratories 32 specimens were used and from one pathological laboratory 40 specimens were used. Whole serial sections were used for the dual-colour CISH and FISH analyses. The specimens were selected, blinded, stained and analysed at each of the five different sites, The specimens were selected based on their HercepTest immunohistochemical (IHC) score, so that 25% in each of the four categories, i.e. 0, 1+, 2+ and 3+, were represented in the study. Therefore, a total of 42 specimens were encompassed in each of the four HER2 IHC categories overall. Of the 168 specimens used, it was impossible to detect tumour cells in only one specimen.

### Fluorescence *in situ* hybridization

FISH was conducted using the *HER2* FISH pharmDx™ (Dako Denmark A/S, Glostrup, Denmark). The assay was performed according to the manufacturer’s recommendations and subsequently evaluated using a fluorescence microscope equipped with appropriate filters for 4′-6-diamidino-2-phenylindole, fluorescein isothiocyanate (FITC) and Texas Red at 60× or 100× magnification.

### Chromogenic *in situ* hybridization

**Figure 1:**
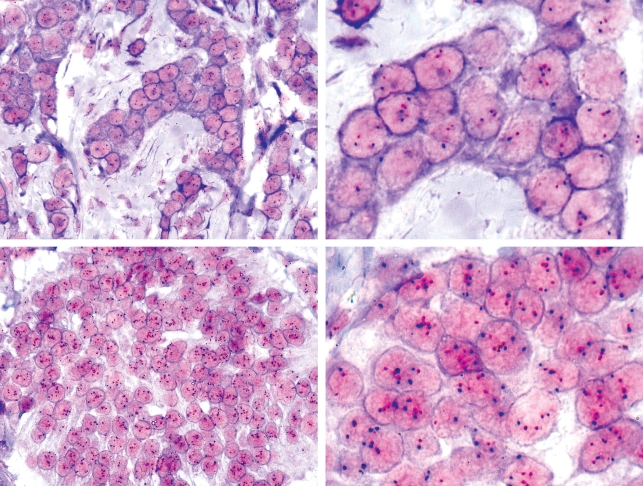
Pictures from two *HER2* non-amplified breast cancer specimens stained with the dual-colour chromogenic *in situ* hybridization protocol. Red dots represent signals from the *HER2* probe, whereas the blue dots represent signals from the CEN-17 reference probe. The upper panel shows a case where tumour cells have a normal number of red and blue signals at 40× (left) and at 100× (right). The lower panel shows a case where tumour cells have a slightly elevated number of both red and blue signals, typical of a non-amplified tumour with chromosome-17 polysomy.

**Figure 2:**
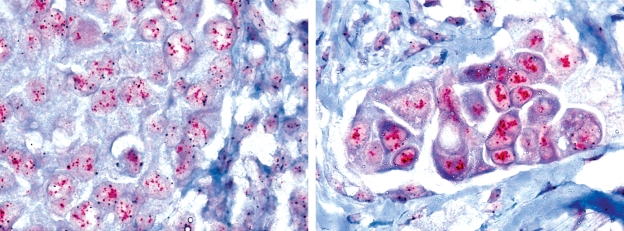
Photomicrographs from two different *HER2* amplified breast cancer specimens stained with the dual-colour chromogenic *in situ* hybridization protocol. In both panels the nuclei of tumour cells show numerous red signals (*HER2*) and a normal number of blue signals (CEN-17). Breast cancer cells with low *HER2* amplification are shown in the left panel, and breast cancer cells with high *HER2* amplification are shown in the right panel. Normal cells can be observed in both panels constituting a very good internal control for the staining of these cases.

The dual-colour CISH protocol used is an extension of the *HER2* FISH pharmDx™ protocol, where the Texas Red- and FITC-labelled FISH probes were visualized using a simple two-step immunohistochemistry staining procedure. The CISH protocol consisted of the same initial steps as in the FISH procedure, with the exception that the final dehydration, air-drying and mounting steps were omitted. Instead, slides were immersed in a wash buffer (50 mmol/l Tris–HCl, 150 mmol/l NaCl, 0.05% Tween 20, pH 7.6) for 3 min. Then excess buffer was tapped away and the slides carefully wiped dry around the sections with a tissue. To block intrinsic peroxidase activity, sections were covered with 200 μl peroxidase block solution (3% H_2_O_2_, 15 mmol/l NaN_3_) for 5 min. Slides were then washed for 3 min in the wash buffer and this washing step was repeated with fresh wash buffer. Excess buffer was tapped off and slides were carefully wiped dry as previously described. To label the FITC- and Texas Red-conjugated FISH probes with HRP- or AP-labelled antibodies, the sections were covered with 200 μl CISH antibody mix containing an HRP-conjugated antibody to FITC and an AP-conjugated antibody to Texas Red in 50 mmol/l Tris buffer, pH 7.5, and incubated for 30 min in a humidified chamber. Slides were washed twice as above in the wash buffer. Excess buffer was tapped off and the slides wiped dry as before. To visualize the AP-conjugated antibodies, sections were covered with 200 μl red chromogen solution (Fast Red KL Salt) and incubated for 10 min in a humid chamber. Slides were washed twice as above. Excess buffer was tapped off and the slides wiped dry as before. In order to visualize the HRP-conjugated antibodies, sections were covered with 200 μl blue chromogen solution (5-amino-2-[3-[5-amino-1,3-dihydro-3,3-dimethyl-1-(4-sulfobutyl)-2H-indol-2-ylidene]-1-propenyl]-3,3-dimethyl-1-(4-sulfobutyl)-3H-Indolium, ter trifluoroacetate) and incubated for 10 min in a humidified chamber. Slides were then washed twice as above. Sections were counterstained with diluted haematoxylin (Dako Denmark A/S; haematoxylin S3301 diluted 1:5 with distilled or deionized water) for 5 min, rinsed with wash buffer and immersed in fresh wash buffer for a minimum of 5 min. Slides were rinsed thoroughly with distilled water and air-dried or dried for 30 min at 37°C. Alternatively, CISH staining was performed on a Dako Autostainer instrument according to the manufacturer’s recommendations. Before mounting of the slides they were cooled to room temperature. They were then mounted with permanent (non-alcohol and non-xylene based) mounting media or aqueous mounting media, and evaluated using a bright field microscope at 40× or 60× magnification. Sections in Figure 2 were photographed using a BX52 microscope (Olympus, Tokyo, Japan) with an UPLAN SAPO 60×/1.2 water immersion lens and a ColorView III camera (Olympus) and processed with Olympus/SIS Cell-F software. Sections in Figure 1 were photographed using a BX51 microscope (Olympus) equipped with a DP70 digital camera (Olympus).

### Scoring of the CISH and FISH slides

Evaluation of the dual-colour CISH and FISH slides was performed blind by the observers and according to the guidelines complementing the Dako *HER2* FISH pharmDx™ kit. One observer at each site scored all local CISH and FISH slides. For all the tumour specimens the *HER2* and centromere 17 (CEN-17) signals from 20 nuclei were counted and the *HER2*:CEN-17 ratios were calculated. If the *HER2*:CEN-17 ratio was borderline (1.8–2.2), another 20 nuclei from this specimen were counted and the *HER2*:CEN-17 ratio was recalculated from all 40 nuclei. These ratios were subsequently converted into a *HER2* gene status of normal or amplified using the cut-off values seen in Table 1. The scoring time, i.e. time used by the observer to score each slide, was recorded using a stopwatch.

**Table 1:** Cut-off values for HER2 status in fluorescence *in situ* hybridization and dual-colour chromogenic *in situ* hybridization

HER2 status	Ratio definitions
Normal	*HER2*:CEN-17 ratio <2.0
Amplified	*HER2*:CEN-17 ratio ≥2.0

### Statistical analyses

Following scoring by CISH and FISH at the local sites, the data were combined. The correlation between dual-colour CISH and FISH results with respect to both gene copy number and ratios for *HER2* and CEN-17 were analysed and the correlation coefficients calculated. Concordance between HER2 status in the dual-colour CISH, FISH and immunohistochemical assays was evaluated by calculating the percent agreement and by κ statistics.^13^ Scoring times of the dual-colour CISH and FISH evaluations were compared using a paired, two-tailed *t*-test. All statistical analyses were performed using SPSS 16 for Windows (SPSS Inc., Chicago, IL, USA).

### Ethics

No data on patient demographics and medical history were collected, and for all study sites this investigation was performed in agreement with local regulations and with the current version of the World Medical Association Declaration of Helsinki.

## Results

### Appearance of the dual-colour CISH in primary breast cancer

Representative photomicrographs of dual-colour CISH-stained primary breast cancer specimens taken at different laboratories are shown in Figures 1 and 2. The dual-colour CISH staining resulted in clear and discernible colours for *HER2* (red) and CEN-17 (blue) that allowed these signals to be counted and quantified. In the upper panel of Figure 1 the majority of tumour cells have nuclei with two red dots representing two *HER2* signals and two blue dots representing two CEN-17 reference probe signals, indicating that these tumour cells do not have *HER2* amplification and have a normal diploid status with respect to chromosome 17. The tumour cells in the lower panel of Figure 1 also represent a *HER2* non-amplified case. In this case most of the nuclei have more than two blue dots, indicating chromosome-17 polysomy. Correspondingly, most tumour cells have nuclei with more than two red dots indicating the presence of extra copies of *HER2* in these tumour cells.

In Figure 2, photomicrographs of amplified tumour cells with many red dots corresponding to several *HER2* signals in the nuclei are observed. Furthermore, the majority of cells have two blue dots per nuclei corresponding to a normal diploid status with respect to chromosome 17. From the photomicrograph in Figure 2 (right panel), the presence of many *HER2* gene copies in the nuclei leads to partially overlapping red signals that can be seen as highly coalescent areas. Although this does not pose a risk for the HER2 status determined for the current specimen, it might lead to a minor underestimation of the *HER2* gene copies present in these tumour cells.

### Concordance between dual-colour CISH and FISH

A HER2 status of normal or amplified was assigned to all breast cancer slides based on the *HER2*:CEN-17 ratio determined in both the dual-colour CISH and FISH protocols. Specimens with a *HER2*:CEN-17 ratio <2.0 were scored as normal, whereas those with a *HER2*:CEN-17 ratio ≥2.0 were scored as amplified. The agreement between *HER2* status when determined by dual-colour CISH and FISH analysis was found to be 100.0% (κ value = 1.00), corresponding to perfect agreement between these two methods (Table 2). Furthermore, to enable comparisons of dual-colour CISH and FISH with the HercepTest IHC score, scores of 0 and 1+ were regarded as negative (normal), whereas a score of 3+ was regarded as positive (amplified). When comparing the IHC HER2 status, without the equivocal IHC 2+ cases, with the status obtained in the dual-colour CISH or FISH protocols, 96.8% agreement (κ value = 0.93) was observed for immunohistochemistry versus dual-colour CISH (Table 3) and for immunohistochemistry versus FISH (Table 4).

**Table 4:** Crosstabulation of HER2 status for immunohistochemistry (IHC) and fluorescence *in situ* hybridization (FISH) without IHC 2+ cases

	HER2 IHC status		
FISH HER2 status	Negative	Positive	Total
Normal	83	3	86
Amplified	1	38	39
Total	84	41	125

Agreement 96.8%.

κ value 0.93.

**Table 3:** Crosstabulation of HER2 status for immunohistochemistry (IHC) and dual-colour chromogenic *in situ* hybridization (CISH) without IHC 2+ cases

	HER2 IHC status		
CISH HER2 status	Negative	Positive	Total
Normal	83	3	86
Amplified	1	38	39
Total	84	41	125

Agreement 96.8%.

κ value 0.93.

**Table 2:** Crosstabulation of HER2 status for fluorescence *in situ* hybridization (FISH) and dual-colour chromogenic *in situ* hybridization (CISH)

	FISH HER2 status		
CISH HER2 status	Normal	Amplified	Total
Normal	122	0	122
Amplified	0	45	45
Total	122	45	167

Agreement 100.0%.

κ value 1.00.

**Figure 3:**
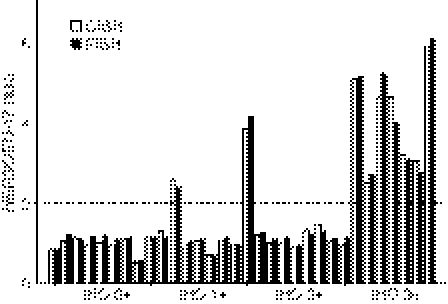
Individual paired *HER2*:CEN-17 ratios for dual-colour chromogenic *in situ* hybridization and fluorescence *in situ* hybridization. Data are from one of the five sites and correspond to 32 individual cases (*n* = 32), with eight cases classified by the HercepTest protocol in each of the four immunohistochemical categories 0+, 1+, 2+ or 3+. The dotted line illustrates the cut-off value of 2.0.

In Figure 3 individual paired *HER2*:CEN-17 ratios determined by the dual-colour CISH and FISH protocols have been graphed from one of the five study sites. Good agreement between the ratios determined by the two methods is obtained, as was observed with the other study sites (data not shown). Figure 3 also shows that from this site one case of IHC 1+ and one case of IHC 2+ were found to be amplified by dual-colour CISH and FISH. This amplified case of IHC 1+ was subsequently re-analysed with HercepTest and verified to be a case of IHC 1+. Taken all sites into account, 7.3% of IHC 3+ cases were found to be normal (negative) by ISH (data not shown), whereas 1.2% of IHC 0 and 1+ cases were found to be amplified. Of all IHC 2+ cases, 14.3% were amplified as determined by the ISH methods. These observations correspond very well to previously reported percentages of amplified cases in the different IHC categories.^14^

**Figure 4:**
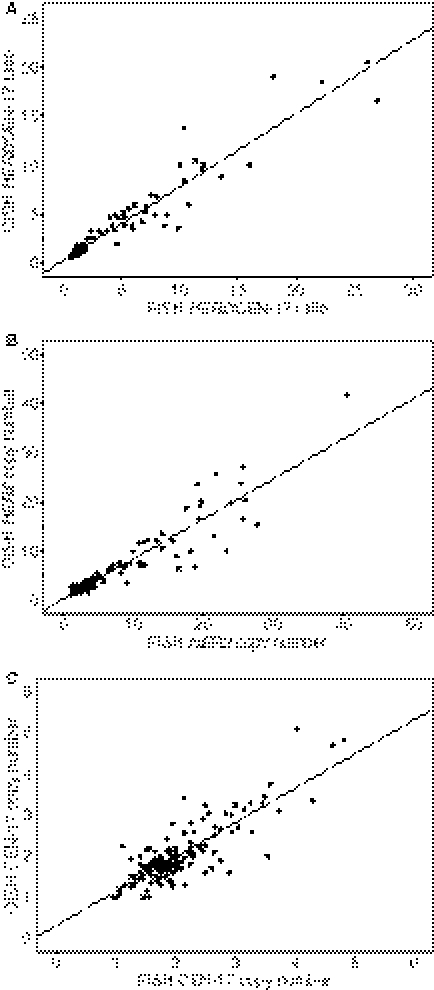
**A**, Correlation between *HER2*:CEN-17 ratios determined by dual-colour chromogenic *in situ* hybridization (CISH) and fluorescence *in situ* hybridization (FISH); *r* = 0.96, *P* < 0.001 (*n* = 167). **B,** Correlation between *HER2* copy numbers determined by dual-colour CISH and FISH; *r* = 0.94, *P* < 0.001 (*n* = 167). **C,** Correlation between CEN-17 copy numbers determined by dual-colour CISH and FISH; *r* = 0.83, *P* < 0.001 (*n* = 167).

Analysis of the correlation between the dual-colour CISH and FISH methods was performed by plotting the *HER2*:CEN-17 ratios, the *HER2* copy numbers and the CEN-17 copy numbers found by the two methods, followed by linear regression analysis (Figure 4). By linear regression analysis the results showed highly significant correlations between the methods with correlation coefficients (ρ) for: (i) *HER2*:CEN-17 ratio at 0.96 (Figure 4A), (ii) *HER2* copy numbers at 0.94 (Figure 4B), and (iii) CEN-17 copy numbers at 0.83 (Figure 4C).

Comparison of the *HER2*:CEN-17 ratios found in the dual-colour CISH and FISH analyses by a paired *t*-test revealed a small but significant difference between these ratios, the mean dual-colour CISH ratio being 0.51 lower than the mean FISH ratio (Table 5). Furthermore, we observed that the mean dual-colour CISH *HER2* copy number was significantly different from the corresponding mean FISH *HER2* copy number in a paired *t*-test, whereas there was no difference between the mean CEN-17 copy numbers determined by FISH and CISH (Table 5).

**Table 5:** Mean *HER2*:CEN-17 ratios and *HER2* and CEN-17 copy numbers for fluorescence *in situ* hybridization (FISH) and dual-colour chromogenic *in situ* hybridization (CISH) for all specimens in the study

	Mean	SEM	SD
FISH *HER2*:CEN-17 ratio	3.08	0.34	4.36
CISH *HER2*:CEN-17 ratio*	2.57	0.26	3.40
FISH *HER2* copy number	5.74	0.54	6.94
CISH *HER2* copy number†	4.88	0.47	6.03
FISH CEN-17 copy number	2.02	0.051	0.66
CISH CEN-17 copy number	2.00	0.051	0.66

Gene copy numbers correspond to the mean copy number per cell of the 20 (or 40) nuclei counted (*n* = 167).

*Significantly different from the FISH *HER2*:CEN-17 ratio (*P* < 0.001, paired two-tailed *t*-test).

†Significantly different from the FISH *HER2* copy number (*P* < 0.001, paired two-tailed *t*-test).

### Scoring time for dual colour CISH and FISH

**Figure 5:**
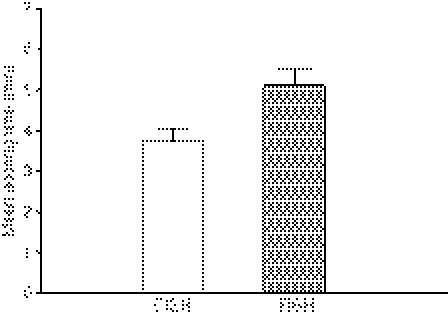
The mean scoring times per slide in the dual-colour chromogenic *in situ* hybridization (CISH) and fluorescence *in situ* hybridization (FISH) analysis. The 95% confidence interval is shown as the error bar. There is a significant 28% difference in mean scoring time between the two methods (*P* < 0.001, two-tailed paired *t*-test, *n* = 167).

To enable comparison of the time used to score slides for the dual-colour CISH and FISH protocols, the time to score each slide with each of the two methods was recorded. From these data we could calculate a mean dual-colour CISH slide scoring time of 3.69 min, and a mean FISH slide scoring time of 5.10 min (Figure 5). Therefore, the dual-colour CISH slide scoring time was found to be 1.41 min shorter (95% confidence interval 1.04, 1.77), which is equal to a significant 28% reduction in scoring time (Figure 5).

## Discussion

A quantitative method for rapid, robust and reliable determination of *HER2* amplification in breast cancer specimens is a clinical requirement. This is because accurate assessment of HER2 status is critical for identification of breast cancer patients that may benefit from treatment with trastuzumab or lapatinib (Tykerb®; GlaxoSmithKline, Brentford, UK),^11^,^15^ and the response to endocrine treatment of metastatic breast cancer also appears to be dependent on HER2 status.^16^,^17^ Determination of HER2 status in breast cancer specimens has previously been performed at the level of *HER2* gene copies using Southern blotting, polymerase chain reaction, FISH or single-colour CISH, or at the level of HER2 protein using immunoblotting, enzyme immunoassays or immunohistochemistry. Today, immunohistochemistry and FISH are the most commonly used clinical methods for determining HER2 status in breast cancer, and these techniques, as well as alternative ISH methods, are currently recommended by the ASCO/CAP.^8^ Generally, FISH analysis is considered the gold standard in HER2 testing;^18^ however, as outlined in the Introduction, there are several limitations and disadvantages of this technique, many of which can be ameliorated by the use of CISH. The use of bright field microscopy instead of fluorescence microscopy in the CISH analysis permits easier identification of invasive tumour cells, reducing the risk of analysing non-malignant or non-invasive cells. Furthermore, the use of bright field microscopy in CISH facilitates the discussion of cases for educational purposes and allows easy evaluation of HER2 status in tissue microarrays prepared for research. One major advantage with the use of CISH is the possibility to store slides in general archives at room temperature, which allows re-evaluation of cases as well as retrospective studies.

Single-colour CISH has previously been documented to be a useful alternative to FISH, and in several studies the concordance levels of HER2 status between single-colour CISH and FISH were found to be in the range of 91–100%.^1^,^2^,^7^,^19^–^26^ However, a single-colour CISH system is limited, as only one type of signal can be evaluated on the section, and HER2 breast cancer specimens that do not have a clear non-amplified or highly amplified HER2 status should be retested on a serial section for a possible chromosome 17 polysomy. This strategy is time consuming and cost ineffective and may delay the final determination of HER2 status.

In this study we have analysed *HER2* amplification in primary breast cancer using a novel, quantitative, dual-colour CISH protocol that converts Texas Red and FITC fluorescent FISH signals to chromogenic red and blue signals, respectively.^27^ In comparison with previous CISH protocols, this dual-colour CISH protocol features simultaneous determination of *HER2* probe signals and chromosome 17 probe signals, thereby eliminating the need for a second round of analysis of chromosome 17 polysomy. In this analysis, we have compared the results of the dual-colour CISH protocol with that of a reference Food and Drug Administration-approved FISH protocol (Dako *HER2* FISH pharmDx™) and, to our knowledge, this is the first multicentre evaluation of the dual-colour CISH kit.

This analysis has revealed a significant correlation of copy numbers for *HER2*, CEN-17 and the *HER2*:CEN-17 ratio between the dual-colour CISH and FISH protocols, which is in agreement with previous reports on the overall good agreement between single-colour CISH and FISH. We here report a 100% concordance in HER2 status between dual-colour CISH and FISH as well as a significant 28% reduction in scoring time when using the dual-colour CISH protocol compared with FISH. Our combined data document that the dual-colour CISH protocol is a reliable and robust analysis method for HER2 testing in breast cancer.

We also observed that the mean dual-colour CISH *HER2* copy number and the mean *HER2*:CEN-17 ratio, but not the mean CISH CEN-17 copy number of the 167 cases were significantly lower than those determined by FISH. This difference might be due to an underestimation of *HER2* copy numbers in amplified cases determined using the dual-colour CISH protocol. As already reported, the number of *HER2* gene copies in the nuclei can be underestimated in areas of high-level amplification due to overlapping dots that lead to coalescing signal clusters (see Figure 2).^19^ This is further supported by the observation that there is no significant difference between the mean *HER2* copy numbers found by the two methods when testing the subset of cases with FISH *HER2* copy numbers ≤2.5 per nucleus (data not shown), whereas when analysing the subset of cases with a FISH *HER2* copy number >2.5 per nucleus, the dual-colour CISH and FISH methods gave significantly different results (data not shown). Because this discrepancy is statistically relevant only at elevated *HER2* copy numbers, there is little risk that this could obscure the *HER2*:CEN-17 ratio of borderline cases, which was also seen by the absence of discordant cases in this study of 167 cases. It should also be noted that a similar trend with dual-colour CISH has been observed previously in a relatively small sample set, and also in this instance this trend did not have any effect on the overall validity of the assay end-point.^27^

We conclude that the dual-colour CISH protocol used in this study is a reliable and robust analysis method that has additional benefits when compared with traditional FISH and single-colour CISH protocols used for HER2 testing in breast cancer.

## Abbreviations:

AP: alkaline phosphatase
ASCO: American Society of Clinical Oncology
CAP: College of American Pathologists
CEN-17: centromere 17
CISH: chromogenic *in situ* hybridization
FISH: fluorescence *in situ* hybridization
FITC: fluorescein isothiocyanate
HER2: human epidermal growth factor receptor 2
HRP: horseradish peroxidase
ISH: *in situ* hybridization
